# New Peptides as Potential Players in the Crosstalk Between the Brain and Obesity, Metabolic and Cardiovascular Diseases

**DOI:** 10.3389/fphys.2021.692642

**Published:** 2021-08-23

**Authors:** Magdalena Czerwińska, Katarzyna Czarzasta, Agnieszka Cudnoch-Jędrzejewska

**Affiliations:** Department of Experimental and Clinical Physiology, Laboratory of Centre for Preclinical Research, Medical University of Warsaw, Warsaw, Poland

**Keywords:** obesity, preptin, adropin, neuropeptide 26RFa, brain feeding areas

## Abstract

According to the World Health Organization report published in 2016, 650 million people worldwide suffer from obesity, almost three times more than in 1975. Obesity is defined as excessive fat accumulation which may impair health with non-communicable diseases such as diabetes, cardiovascular diseases (hypertension, coronary artery disease, stroke), and some cancers. Despite medical advances, cardiovascular complications are still the leading causes of death arising from obesity. Excessive fat accumulation is caused by the imbalance between energy intake and expenditure. The pathogenesis of this process is complex and not fully understood, but current research is focused on the role of the complex crosstalk between the central nervous system (CNS), neuroendocrine and immune system including the autonomic nervous system, adipose tissue, digestive and cardiovascular systems. Additionally, special attention has been paid to newly discovered substances: neuropeptide 26RFa, preptin, and adropin. It was shown that the above peptides are synthesized both in numerous structures of the CNS and in many peripheral organs and tissues, such as the heart, adipose tissue, and the gastrointestinal tract. Recently, particular attention has been paid to the role of the presented peptides in the pathogenesis of obesity, metabolic and cardiovascular system diseases. This review summarizes the role of newly investigated peptides in the crosstalk between brain and peripheral organs in the pathogenesis of obesity, metabolic, and cardiovascular diseases.

## Introduction

Obesity, according to the American Association of Clinical Endocrinologists and the American College of Endocrinology, is a chronic condition resulting in deposits of excessive fat tissue in an organism leading to increased morbidity and mortality (Garvey et al., 2014). The World Health Organization (WHO) reported in 2016 that the obese population has nearly tripled since 1975 and accounts for more than 650 million (13%) adults worldwide (World Heatlh Organisation., 2016). Moreover, the prevalence of obesity in children aged 5–19 is estimated to be over 124 million, which means that the number of obese children has rocketed from less than 1% to 6%–8% from 1975 to 2016 (World Heatlh Organisation., 2016). It has been proven that childhood obesity promotes adult obesity in the future (World Heatlh Organisation., 2016). If these secular trends continue then by 2030 an estimated 20% of the world’s adult population will be obese (Kelly et al., 2008), despite many preventive interventions by the WHO.

The decisive cause of obesity is a visible disproportion between caloric input and output (Garvey et al., 2014; Mohammed et al., 2018). This stems from the complex interaction of biological (genetics), environmental, and socioeconomic factors, as well as behavioral factors (Garvey et al., 2014). These factors interrelate with overlapping or additional subsets of gene-environment interactions, which determines the severity of the disease, and thus affecting the health condition and the manifestation of accompanying diseases called complications (Garvey et al., 2014). Recently, special attention has been given to the role of the gut-brain axis (GBA) in the pathogenesis of obesity (le Roux et al., 2007).

Obesity-related complications include various diseases affecting every body system, starting from the cardiovascular, gastrointestinal (GI), endocrinological, and musculoskeletal systems (Garvey et al., 2014; World Heatlh Organisation., 2016), and even including psychiatric disorders, infertility, and 13 excess body fat related cancers (Lauby-Secretan et al., 2016). Cardiovascular diseases such as hypertension, coronary artery disease, heart failure, and arterial fibrillation in the course of obesity appear to be crucial complications affecting longevity (Lavie et al., 2009). Despite medical development in diagnostic tools and treatment, the dominant causes of death in the obese are cardiovascular events (Afshin et al., 2017). It is believed that most of the above complications can be alleviated or treated by losing weight, and weight maintenance at a plateau level is challenging (Bischoff and Schweinlin, 2020). Weight status related to energy homeostasis, appetite control, and nutrient oxidation is controlled by the cooperation of the nervous and endocrine systems (Ghanemi et al., 2018).

## Mechanism of Energy Balance and Appetite Control

Energy homeostasis, appetite, and body weight are primarily controlled by the central nervous system (CNS) orexigenic and anorexigenic neurons, which connect the peripheral organs such as the gastrointestinal tract, the pancreas, and the adipose tissue in a complex manner (Gadde et al., 2018). Signals concerning food consumption and energy status of the organism create long-term (adiposity by leptin and insulin) energy responses or short-term (hunger, satiation, and satiety mostly by GI hormones) energy responses (Bauer et al., 2016). Peripheral information through the circulation or nerves affects these CNS areas, stimulating or suppressing specific neurons to regulate the energy balance with the use of neuronal and endocrinal responses (Wilson and Enriori, 2015; Bauer et al., 2016).

The most important structure of the CNS playing a key role in the central control of food intake is the hypothalamus, especially the arcuate nucleus (Arc), the paraventricular nucleus (PVN), the ventromedial hypothalamus (VMH), and the lateral hypothalamic area (LHA) (Timper and Brüning, 2017). It has been proven that the above nuclei of the hypothalamus form interconnected neural loops, sending projections to the brainstem as well as to the higher centers of the brain to maintain an organism’s energy homeostasis (Roh and Kim, 2016; Abdalla, 2017).

The Arc expresses receptors for ghrelin, insulin, leptin, and nutrient sensors (Wilson and Enriori, 2015; Sutton et al., 2016). The presence of the above receptors makes the Arc the first hypothalamic area that senses hormonal and nutrient signals (Wilson and Enriori, 2015; Sutton et al., 2016). Peripheral hormones such as leptin and insulin, etc., act on the hypothalamus and regulate satiety and the glucose-lipid metabolism in response to food digestion (Hutch and Sandoval, 2017). The Arc contains two functionally opposite subpopulations of neurons, enabling crosstalk between the hypothalamus nuclei, the brainstem centers, and the peripheral organ substances (Timper and Brüning, 2017). One orexigenic neuron subpopulation secretes neuropeptide Y (NPY) and agouti-related peptide (AgRP), while the other subpopulation produces anorexigenic proopiomelanocortin (POMC) and cocaine and amphetamine regulated transcript (CART) (Schalla et al., 2021). NPY through the Y1 and Y5 receptors and AgRP as an antagonist of α-Melanocyte-stimulating hormone (α-MSH) stimulate the appetite and decrease energy expenditure (Clark et al., 1984; Rossi et al., 1998; Yulyaningsih et al., 2011). POMC releases α-MSH, an agonist of the melanocortin 4 receptor (MC4R), which decreases food consumption and increases energy use (Fan et al., 1997; Abbott et al., 2000). The NPY/AgRP and POMC/CART neurons are defined as first-order neurons in response to peripheral and brain inputs and project to second-order neurons located in other hypothalamic nuclei (PVN, VMH, LHA, DMH) or the brainstem (Roh and Kim, 2016; Schalla et al., 2021).

The PVN integrates signals from the Arc, the LHA, the VMH, and the brainstem (López et al., 2007; Sutton et al., 2016), and contains anorexigenic neurons producing oxytocin, corticotropin- (CRH), and thyrotropin-releasing hormone (TRH) (Abdalla, 2017). Orexin neurons also mediate the vast majority of hypothalamic output, regulating food consumption and energy use (Sutton et al., 2016). Similar to the Arc, satiety/hunger signals reach the PVN (Wilson and Enriori, 2015). Furthermore, injury to the PVN has been proven to result in hyperphagia and obesity (Leibowitz et al., 1981). Additionally, the PVN contains pre-autonomic neurons influencing the lower autonomic centers, which control autonomic metabolism (Yi and Tschöp, 2012). The PVN also interferes in the endocrine secretion of the thyroid and the adrenal gland (Yi and Tschöp, 2012).

The VMH expresses the anorexigenic brain-derived neurotrophic factor (activated by the POMC/CART neurons of the Arc) and MC4R, the leptin receptor (Xu et al., 2003). VMH destruction leads to overeating and then to obesity (Shimizu et al., 1987).

The LHA is considered to mediate the motivational processes underlying feeding and other goal-directed behaviors necessary for survival, including translating motivation into action (Petrovich, 2018). The LHA expressing orexigenic melanin-concerning hormone and orexin (also known as hypocretin) and is influenced by vagal input and the reward system (Moehlecke et al., 2016).

Expecting input from the hypothalamic nuclei, the brainstem is another key region in the brain, regulating food and energy homeostasis (Prinz and Stengel, 2017). The dorsal vagal complex (DVC) is located in the brainstem and includes the nucleus of tractus solitarius (NTS), the dorsal nucleus of the vagus nerve (DMV), and the area postrema (AP). The DVC is crucial in the analysis and transportation of mechano- and chemosignals from the intestines to the hypothalamus (Bailey, 2008). Not only do cholecystokinin (CCK) and ghrelin affect the NTS through the vagal nerve, but also *via* the circulation through the area postrema (Bauer et al., 2016). A vast range of receptors have been detected for hormones controlling food consumption in the vagal afferent neurons of the brainstem: CCK-1R and 2R for CCK and gastrin (Moriarty et al., 1997), leptin (Burdyga et al., 2002), insulin, glucagon-like peptide-1 receptor (GLP-1R) (Nakagawa et al., 2004), glucagon-like peptide-2 receptor (GLP-2R) (Nelson et al., 2007), and orexin receptor called hypocretin receptor 1 (HCRTR1) (Burdyga et al., 2003). The NTS expresses Y1, Y5, and MC4R and produces glucagon-like peptide-1 (GLP-1), NPY, POMC-derived α-MSH (these POMC neurons are important for short-term regulation) (Roh and Kim, 2016). Brainstem cells are involved in the motor output of the neuronal circuitry controlling feeding behaviors, which indicates a gut-brain (stem) role in the energy balance by alternating energy expenditure.

The hypothalamus and the brainstem create circuits with the autonomic nervous system (ANS), which regulates energy expenditure to control peripheral metabolism in white and brown adipose tissue, the gastrointestinal tract, and the skeletal muscles (Seoane-Collazo et al., 2015; Imai and Katagiri, 2021). The ANS regulates pancreatic endocrine activity, hepatic- and tissue-specific insulin sensitivity, lipid mobilization in white adipose tissue (WAT), and the glucose process (Imai and Katagiri, 2021).

Special attention has recently been given to the role of the gut-brain axis (GBA) in the regulation of energy balance and appetite control (le Roux et al., 2007). The GBA is a bidirectional signaling pathway between the gut and the CNS aimed at maintaining the energy balance (Bauer et al., 2016). Information regarding meal size and the caloric and nutrient composition from various locations in the gastrointestinal tract is converted, mainly, into neuronal and hormonal signals which reach the CNS, and by means of its response enables the achievement of an energy balance in the fasting and feeding conditions (Bauer et al., 2016). These signals can be projected to the CNS *via* paracrine vagal and spinal transmission or through the endocrine way (Wilson and Enriori, 2015; Bauer et al., 2016). Finally, the vagal and spinal afferent neurons concentrate the hormonal and mechanical stimuli into the NTS, which integrates and projects them into the hypothalamus (Sobrino Crespo et al., 2014; Bauer et al., 2016). Signal transmission in the NTS is mediated by POMC, catecholaminergic neurons, and *N*-methyl-D-aspartate (NMDA) receptors (Bauer et al., 2016; Bliss and Whiteside, 2018). In addition to the NTS, the Arc is also involved in the GBA, which responds to peripheral and central appetite signals *via* POMC and AgRP (Bliss and Whiteside, 2018). Located in the medial Arc subpopulation of AgRP neurons, releasing two inhibitory neurotransmitters- AgRP and NPY, that stimulate hunger and appetite and decrease energy expenditure, thereby contributing to excessive food consumption and weight gain (Clark et al., 1984; Abbott et al., 2000; Yulyaningsih et al., 2011).

The CNS coordinates intestinal activities through the sympathetic and parasympathetic branches of the ANS and the hypothalamic-pituitary-adrenal axis (HPA) (Yi and Tschöp, 2012).

Enteroendocrine cells (EECs) located in the intestinal epithelium synthesize and secrete gut peptides such as CCK, peptide YY (PYY), GLP-1, oxyntomodulin (OXM), and ghrelin (Adriaenssens et al., 2018). Afferent vagal nerves terminating close to the basolateral part of the EECs are enriched by ghrelin, leptin, CCK, and GLP-1 receptors, which are highly activated during digestion (Gribble and Reimann, 2016). GI peptides reach the hypothalamus not only by the influence of vagal transmission but also *via* systemic circulation through the Arc or AP (Bauer et al., 2016). GI hormones can indirectly activate spinal and vagal afferent neurons *via* activation of neurons of the enteric nervous system, which has also been shown to express gut peptide receptors (Richards et al., 2014; Bauer et al., 2016).

GI hormones are divided into two main groups related to their effect on food intake: (i) preprandial ghrelin or endocannabinoids have an orexigenic effect; (ii) during and after the prandial period—insulin, leptin, CCK, PPY, GLP-1 and 2, OXM, pancreatic polipeptide, gastrin, neurotensin, and also stomach distension result in anorexigenic activity (Bellocchio et al., 2008; Perry and Wang, 2012).

Numerous results discussed in the current manuscript illustrate the influence of selected peptide hormones on pancreatic beta cells and their function. The pancreas consists of numerous pancreatic islets containing different cells distinguished by their primary endocrine hormone products: α-cells (glucagon), β-cells (insulin and amylin), δ-cells (somatostatin), γ-cells (pancreatic polypeptide), and rare ε-cells (ghrelin) (Campbell and Newgard, 2021). These signals like GI hormones regulate hunger, satiety, energy homeostasis, and body weight (Campbell and Newgard, 2021). Especially, insulin—an anabolic peptide released in bi-phasic pattern related to glucose serum levels, regulate glycemia by promoting glucose uptake and storage mostly in muscles and adipose tissue (Plum et al., 2006; Loh et al., 2017; Campbell and Newgard, 2021). Moreover, insulin secreted in direct proportion to visceral fat mass is one of the adiposity signals that take part in long-term satiety regulation (Woods et al., 2006; Roh and Kim, 2016). Insulin also leads to decreased food consumption *via* inhibition of NPY/AgRP and stimulation of POMC/CART hypothalamic neurons mostly in Arc (López et al., 2007; Loh et al., 2017).

## Metabolic and Cardiovascular Consequences of Obesity

In the course of obesity, changes in the composition and activity of adipose tissue (adiposopathy) are observed, leading to the development of metabolic disorders (Blüher, 2013). The most common metabolic disorder associated with obesity is insulin resistance/hyperinsulinemia, which in turn leads to metabolic dyslipidemia (Klop et al., 2013). During obesity, adipocytes become insulin resistant, which stimulates lipolysis processes in them and the release of excessive amounts of free fatty acids into the circulation, which have a lipotoxic effect on non-adipose tissues, including the cardiovascular system (Boden and Shulman, 2002). A large body of clinical and epidemiological evidence point to obesity as an independent risk factor for cardiovascular diseases (CVD), including coronary artery disease (CAD), heart failure (HF), hypertension, cerebrovascular disease, atrial fibrillation (AF), ventricular arrhythmias, and sudden cardiac death (Poirier et al., 2006; Dwivedi et al., 2020).

Obesity causes a number of abnormal adaptations in the structure and functioning of the cardiovascular system, such as: 1) an increase in total circulating blood volume due to sodium retention; 2) increased cardiac output (CO) through an increase in stroke volume (SV) and a mild increase in heart rate due to sympathetic activation; 3) increased vascular resistance (SVR) due to low-grade inflammation, hyperinsulinemia, an overactive sympathetic nervous system, and sleep-disordered breathing (Johnson et al., 1975; Alpert, 2001). The increase in CO and SVR causes higher blood pressure, consequently leading to hypertension (Messerli et al., 1982). The above hemodynamic changes lead to an increase in the workload of the heart and predispose obese people to remodel the left ventricle (LV) (Alpert, 2001). Eccentric or concentric LV hypertrophy is then observed, leading to an initial progressive diastolic and ultimately systolic LV dysfunction (Alpert et al., 2016). Obesity-related myocardial dysfunction is known as obesity-associated cardiomiopathy (adipositas cordis) and refers to the gradual replacement of cardiomyocytes with bands of adipose tissue (Sletten et al., 2018). Combined with the toxic effects of locally released adipokines (biologically active substances synthesized by the adipose tissue) on the adjacent heart muscle, this fat infiltration may induce lipotoxicity and may promote cardiomyocyte dysfunction associated with myocardial hypertrophy and fibrosis (Roberts and Khan, 2020). It is believed that epicardial adipose tissue is of key importance for the development of obesity-related cardiovascular complications (Guglielmi and Sbraccia, 2017).

In the present review, we focused on the pleiotropic effect of 26/43RFa, preptin, and adropin and we highlight the underlying mechanism discovered by research in recent years. Gaps in knowledge are also mentioned to indicate the further direction of studies. A PubMed search was performed for all articles with the listed peptides.

## Neuropeptide 26RFa

Neuropeptide 26RFa is a 26-amino acid peptide also known as N-terminal extended form 43RFa or QRFP (from pyroglutamylated arginine-phenylalanine-amide peptide) together known as the QRFP system (Chartrel et al., 2003; Fukusumi et al., 2003; Jiang et al., 2003). It was first detected in the frog brain (Chartrel et al., 2003) and rat brain (Fukusumi et al., 2003) but subsequently was confirmed in humans (Chartrel et al., 2003; Bruzzone et al., 2006). In mice and rats, these neurons are located in the VMH, the LHA, and the Arc (Chartrel et al., 2003; Kampe et al., 2006) but peripherally are expanded differently compared with humans (Fukusumi et al., 2003; Jiang et al., 2003) (Table 1). 26RFa genes are expressed in the human hypothalamus, specifically in the PVN, the VMH, and the spinal cord, mostly in the dorsal horn (Bruzzone et al., 2006). They are also distributed peripherally in the endocrine glands, coronary artery, stomach (Prévost et al., 2015), intestine, bladder, prostate (Jiang et al., 2003), and pancreas (Prévost et al., 2015). 26/43RFa acts through the orphan receptor GPR103 (Jiang et al., 2003), which is a 7-transmembrane G protein-coupled receptor (GPCR). GPR103 is expressed in the human brain, especially in the cerebral cortex, hypothalamus, vestibular nuclei, hippocampus, and amygdala (Jiang et al., 2003), and peripherally in the heart, glands, kidneys, testes (Jiang et al., 2003), and pancreas (Granata et al., 2014; Prévost et al., 2015). In rodents, the analog of GPR103, the QRFP-receptor exists in two isoforms (QRFP1, QRFP2) (Kampe et al., 2006; Takayasu et al., 2006), and is highly expressed in the brain (Bruzzone et al., 2007) and also peripherally, for example, in the adipose tissue, the skeletal muscles, and the pancreas.

**TABLE 1:** Expression of the 26RFa neuropeptide and GPR103 in humans and rodents.

	**Expression in human**		**Expression in rat**		**Expression in mice**	
	**Peptide**	**Receptor**	**Peptide**	**Receptor**	**Peptide**	**Receptor**
Nervous system	PVN, VMH, spinal cord (the dorsal and lateral horn)	cerebral cortex, hypothalamus, thalamus, vestibular nucleus, ganglion, amygdala, caudate nucleus, hippocampus, dorsal horn of spinal cord, pituitary gland	VMH, Arc, LHA, retrochiasmatic area, pituitary	VMH, LHA, PVN, Arc, piriform cortex, amygdalohippocampal area, lateral septum, reuniens thalamic nucleus, nucleus of the solitary tract, raphe nuclei, locus coeruleus, zona incerta, intergeniculate leaf, medial parabrachial nucleus, dorsal horn of the spinal cord, hippocampus, reuniens thalamic nuclei, parafascicular thalamic nuclei, amygdala, pituitary, the vestibular nucleus, medial geniculate, interpeduncular nucleus, median preoptic area, paragigantocellular nucleus, facial and hypoglossal nuclei More detailed GPR103 brain expression is available in articles: (Kampe et al., 2006; Bruzzone et al., 2007).	PVN, LHA	mitral cell layer of the olfactory bulb, island of Calleja, solitary tract, caudate putamen, olfactory tubercle, triangular septal nucleus, suprachiasmatic nucleus, PVN, magnocellular nucleus of the hypothalamus, retroventrolateral reticular nucleus, medial supramammillary nucleus and facial nucleus, spine
Peripheral tissues	Pituitary gland, thyroid glands, parathyroid glands, testes, coronary artery, stomach, duodenum, ileum, colon, bladder, prostate, adrenal glands, pancreas	fetal bone, osteoblasts, heart, thyroid glands, parathyroid glands, kidneys, testes, stomach, duodenum, ileum, colon, pancreas, adrenal glands	Eyes, trachea, mammary glands, testes, thymus, salivary glands, uterus, pancreas, skeletal muscle, adrenal glands	Eyes, testes, adrenal glands, kidneys, pancreas, adipose tissue, skeletal muscles	Eyes, trachea, mammary glands, testes, thymus, salivary glands, uterus, adipose tissue, pancreas, duodenum, jejunum, ileum	Eyes, the thymus, adrenal glands, testes, striated muscles, liver, adipose tissue

*Arc, arcuate nucleus; LHA, lateral hypothalamic area; PVN, paraventricular nucleus; VMH, ventromedial hypothalamus.*

Recently, the QRFP system is a popular target for research on metabolic homeostasis, glucose and lipid metabolism, pancreas function, and obesity (Mulumba et al., 2010, 2015; Méquinion et al., 2017; Prévost et al., 2019a, b; El-Mehdi et al., 2020).

### Effect on Energy Homeostasis and Feeding

**FIGURE 1:**
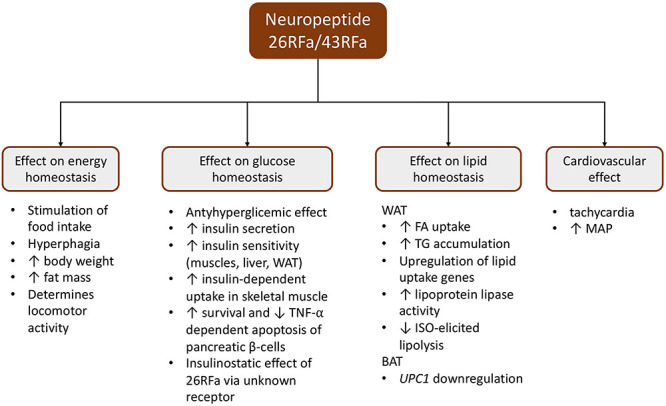
Schematic presentation of the pleiotropic effect of the 26RFa/43RFa system. BAT, brown adipose tissue; ISO, isoproterenol; MAP, mean arterial pressure; TG, triglyceride; TNF-α, tumor necrosis factor α; WAT, white adipose tissue; UPC1, uncoupling protein 1.

The influence of the QRFP system on food intake has been investigated since being detected in the hypothalamic regions regulating feeding habits and appetite (Fukusumi et al., 2003) (Figure 1).

Central administration of 26RFa/43RFa revealed orexigenic activity. Numerous studies have demonstrated that acute intracerebroventricular (ICV) 26RFa injection resulted in a dose-dependent increase in food intake in fasted mice and rats (Messerli et al., 1982; Fukusumi et al., 2003; Kampe et al., 2006). Additionally, 43RFa showed a greater stimulation in feeding behavior than 26RFa in ob/ob and db/db C57BL/6J (B6) mice (Fukusumi et al., 2003). Moreover, chronic (13 days) ICV 43RFa infusion generated significant body and fat mass gain, as well as hyperphagia in B6 mice maintained on a moderately high-fat diet (Prévost et al., 2015). It has been proven that the orexigenic activity of the QRFP system was observed in both mice and rats on a standard fat diet, a moderately high-fat diet, and a high-fat diet (Takayasu et al., 2006; Granata et al., 2014; Prévost et al., 2015; Guglielmi and Sbraccia, 2017).

Studies in transgenic animals provided additional information in understanding the effects of the QRFP system on energy homeostasis and food intake. QRFP^–/–^ B6 mice (eGFP knock-in; 26RFa-KO) fed on a standard and a high-fat diet were thin and hypophagic (Prévost et al., 2019b). In contrast, 26RFa^–/–^ B6 mice fed on a standard chow diet did not reveal changes in body weight, fat mass, food intake, compared with 26RFa^+/−^ and 26RFa^+/+^ (Prévost et al., 2019a).

Furthermore, the available data indicate that the diet macronutrient profile has an impact on preproQRFP gene expression in the hypothalamus. Primeaux (Takayasu et al., 2006) has shown that, after 3 weeks on a high-fat (55%) diet, consumption increased the concentration of preproQRFP mRNA in the VHM/ARC, but not in the LHA. In contrast, Beck et al. (El-Mehdi et al., 2020) have shown that the expression of 26RFa/QRFP was undetectable in Long-Evans rats fed on a high-fat diet, yet in the control group (30% fat) the expression was lower than in the low-fat (5%) diet group. Nevertheless, the application of a caloric restriction diet for 10 weeks in B6 mice resulted in no changes in the expression of 26RFa and GPR103 (Méquinion et al., 2017). Moreover, 3 days of pharmacological oral treatment with GPR103 antagonist e25 (Mulumba et al., 2010, 2015) in diet-induced obesity (DIO) B6 mice decreased food intake in a dose-dependent manner without changing the feeding pattern, but with a noticeable start in weight reduction (Mulumba et al., 2010, 2015).

Clinical studies also revealed higher plasma concentrations of the 26RFa peptide in obese patients and patients with type 2 diabetes compared with healthy subjects (Prévost et al., 2015, 2019b). However, studies by Prévost et al. (2015) found no correlation between plasma 26RFa concentration and age, body mass index (BMI), waist circumference, and fasting blood glucose. On the other hand, they found a positive correlation between the plasma concentration of 26RFa and fasting plasma insulin and the insulin resistance marker HOMA-IR (Prévost et al., 2015). In turn, further studies also showed a higher plasma concentration of 26RFa in obese patients, which correlated positively with BMI and with various parameters of insulin secretion and insulin resistance (Prévost et al., 2019b). Additional information on the regulation of 26RFa release into the circulation has been provided by clinical studies conducted by Galusca (Galusca et al., 2012). These researchers showed a higher concentration of 26RFa in malnourished women suffering from anorexia nervosa compared with healthy women. Additionally, each meal resulted in a delayed reduction of 26RFa plasma concentration in anorectic women, which returned to the mean level before the next meal (Galusca et al., 2012). This suggests an adaptive mechanism of prolonged activation of the 26RFa system in response to a chronic reduction in calorie intake and energy storage. This research also provided evidence on the QRFP circadian rhythm with its highest level in the morning (10 am), falling through the afternoon and evening, and reaching its lowest level at midnight, which was disturbing for anorectic women (Galusca et al., 2012).

### Effect on Glucose Homeostasis

The first study linking 26RFa glucose and homeostasis revealed that a 26RFa dose-dependent reduction in glucose, arginine, and exendin-4 induced insulin secretion in Wistar rats, but neither affected the basal insulin level nor the glucagon level (Egido et al., 2007). Further analysis uncovered 26/43RFa and GPR103 expression in INS-1E rat β-cells and mouse insulinoma cells (MIN6), as well as human pancreas islet cells (Granata et al., 2014; Prévost et al., 2015).

Granata et al. (2014) discovered that 26/43RFa promotes survival and prevents apoptosis caused by starvation, cytokines, and glucolipotoxicity in human pancreatic islets and INS-1E rat β-cells, *via* the ERK1/2 and PI3K/Akt signaling pathways. Furthermore, during glucose overload (Granata et al., 2014), 26RFa has an insulinostatic (Bruzzone et al., 2006; Georgsson et al., 2015) and an insulinotropic effect, while 43RFa only has an insulinotropic effect (Bruzzone et al., 2006). Moreover, 43RFa caused an increase in basal insulin plasma concentration independent of glucose stimulation and also caused an increase in glucose uptake (Granata et al., 2014).

It has been shown that 26RFa has an antihyperglycemic effect. Intraperitoneal (i.p.) injection of 26RFa during glucose tolerance tests in mice reduced glucose-induced hyperglycemia by the enhancement of insulin synthesis and targeted tissue insulin sensitization (co-expression of GPR103, glucose transporter type 4, and insulin receptor in the muscles, liver, and adipose tissues) (Prévost et al., 2015). Diet-induced chronically obese B6 mice lost the antihyperglycemic effect of 26RFa as a result of the abolition of the neuropeptide’s impact on insulin secretion, the depletion of insulin sensitivity, and mRNA downregulation related to GPR103 in the pancreatic islets, the adipose cells, and the muscle cells (Prévost et al., 2019a). Additionally, the application of a GPR103 antagonist during an oral glucose test in B6 mice on a standard chow diet resulted in severe hyperglycemia with significant insulin sensitivity reduction (Prévost et al., 2019a). Whereas, the administration of a GPR103 antagonist in DIO B6 mice did not alter the glycemic response in the glucose tolerance test (Prévost et al., 2019a).

Studies in transgenic mice confirmed the key role of 26RFa in glucose homeostasis. The model of mice deficient in the 26RFa gene (26RFa^–/–^ B6; 26RFa-KO) was characterized by lower basal insulin levels and higher glucose-stimulated hyperglycemia with impairment in insulin synthesis and unaltered insulin sensitivity (El-Mehdi et al., 2020). Furthermore, 26RFa-KO mice, in fasting, revealed an overregulation of glucose-6-phosphatase (G6PC) and phosphoenolpyruvate carboxykinase 1 (PCK1) promoting gluconeogenesis with a slight reduction in glucokinase (GCK) enhancing glycogen accumulation (El-Mehdi et al., 2020). The pancreases of 26RFa deficient B6 mice were larger with more fat storage, with a higher number of islet areas, and β-cells containing less insulin than the control group (El-Mehdi et al., 2020).

Clinical research has identified a positive correlation between the elevated plasma 26RFa, the fasting insulin level, and the insulin resistance marker—Homeostatic Model Assessment of Insulin Resistance (HOMA-IR) in diabetic and obese patients (Prévost et al., 2015, 2019b). In healthy participants, 26RFa was stable for the first 90 min but was elevated 120 min after the oral glucose tolerance test (OGTT) (Prévost et al., 2015). The 26RFa peak was delayed in patients with gastroparesis, whereas the peak appeared earlier in patients following a gastrectomy (Prévost et al., 2015). A higher plasma 26RFa level after glucose overload suggests its production in the GI tract and activity as incretin. During the OGTT test in the obese, 26RFa concentration was significantly increased, but its concentration did not fluctuate as in the case of healthy people (Prévost et al., 2019b). Additionally, the plasma 26RFa concentration revealed a positive correlation between the body mass index, HOMA-IR, and fasting insulinemia (Prévost et al., 2019b).

### Effect on Lipid Homeostasis

Experimental studies have shown the presence of 26/43RFa and GPR103b mRNA and protein in adipose 3T3-L1 cells and the fat tissue of DIO C57BL/6 mice (Mulumba et al., 2010). However, DIO C57BL/6 mice presented inactive preproQRFP mRNA and an increased GPR103 type 2 receptor mRNA level in epididymal adipose tissues (Prévost et al., 2019b). In addition, it was shown that 26/43RFa are involved in the increase in fatty acid uptake, triglyceride accumulation, lipoprotein lipase activity, and upregulation of lipid uptake genes (long-chain fatty acid transport protein 1, FATP1; cluster of differentiation 36, CD36; lipoprotein lipase, LPL; long-chain-fatty-acid-CoA ligase 1, ACSL1; peroxisome proliferator-activated receptor gamma, PPAR-γ; and CCAAT/enhancer-binding protein alpha, C/ebpα) (Mulumba et al., 2010, 2015). Moreover, studies on 3T3-L1 cells and omental human adipocytes revealed that QRFP43 may inhibit isoproterenol (ISO)-elicited lipolysis *via* activation of the PI3K/Akt/phosphodiesterase 3B pathway (Mulumba et al., 2010, 2015).

### Cardiovascular Effect

The available data indicate that 26RFa does not appear to have an effect on myocyte contractile function (Nichols et al., 2010). Nevertheless, Takayasu et al. (2006) noticed that ICV injection in aware B6 mice had an effect on the mean arterial pressure (MAP) and the heart rate (HR). Additionally, Fang et al. (2009) showed that intravenous (i.v.) administration of 26RFa in anesthetized Wistar rats revealed a short persistent (about 3 min) biphasic reaction of the MAP (from hypotension to hypertension) with accompanying tachycardia (Fang et al., 2009). Moreover, the same researchers observed that the pressor effect of 26RFa is different when using other isoforms of this protein (Fang et al., 2009).

### Neuropeptide 26RFa and the Gut-Brain Axis

Evidence for the participation of 26/43RFa in the gut-brain axis is provided by studies confirming the presence of the expression of this peptide in the hypothalamic neurons regulating food intake, such as: Arc, LHA, and VMA (Fukusumi et al., 2003; Jiang et al., 2003; Timper and Brüning, 2017). Moreover, the expression of GPR103 was detected in many structures of the CNS, including the hypothalamus (Jiang et al., 2003). It appears that 26/43RFa may be involved in the central regulation of food intake through the gut-brain axis *via* neuropeptide Y and POMC neurons in the Arc (Takayasu et al., 2006; Lectez et al., 2009). It has been shown that ICV 26RFa infusion can stimulate preproNPY mRNA expression and NPY release, and can stimulate a decrease in POMC expression and α-MSH levels in the hypothalamus, as well as stimulating food consumption (Lectez et al., 2009). Additionally, it has been described that POMC neurons lacking GPR103 receptors are stimulated by 26RFa *via* the NPY neurons which have GPR103 receptors (Lectez et al., 2009). Interestingly, the administration of 26RFa together with antagonists suppressing the activity of neuropeptide Y, Y1, and Y5 receptors not only abolished the activity of these receptors but also reduced food consumption (Takayasu et al., 2006; Lectez et al., 2009; Zagorácz et al., 2015). Moreover, the available data indicate that diet composition influences the expression of the preproQRFP gene in the nucleus of the hypothalamus (Primeaux et al., 2008; Beck and Richy, 2009; Georgsson et al., 2014, 2015; Méquinion et al., 2017).

However, it has also been shown that 26RFa and its GPR103 receptor are expressed in peripheral organs and tissues, including the gastrointestinal tract: stomach, intestines, and pancreas, as well as in the adipose tissue, heart, kidneys, and endocrine glands (Takayasu et al., 2006; Mulumba et al., 2010; Prévost et al., 2015). Therefore, the above data suggest that 26RFa may also be involved in the regulation of peripheral ingestion and metabolic processes such as: stimulation of fatty acid uptake, triglyceride accumulation, and an increase in lipoprotein lipase activity in adipocytes (Mulumba et al., 2010, 2015).

## Preptin

Preptin is a 34-amino-acid peptide from the insulin family (Wallis, 2009; Cheng et al., 2012) derived from proinsulin-like growth factor II (proIGF-II) and is co-secreted with insulin, amylin, and pancreostatin from β-cells (Buchanan et al., 2001). Recent research detected preptin mRNA in the liver, heart, and skeletal muscles of rats (Moustafa and Arisha, 2020) (Table 2). In humans, preptin is presented in the pancreas (Höög et al., 1993), kidneys (Buchanan et al., 2001), and mammary tissue (Aydin et al., 2013a). Based on research by Cheng (Cheng et al., 2012), it is supposed that preptin acts *via* the IGF-II receptor, but a receptor dedicated to preptin has not been yet indicated.

**TABLE 2:** Expression of the preptin and preptin receptor in humans and rodents.

	**Expression in human**		**Expression in rodent**	

	**Peptide**	**Receptor**	**Peptide**	**Receptor**
Preptin	Pancreas- B cells, liver, heart, skeletal muscles	Not known*	Pancreas, kidney mammary tissue	Not known*

*It is supposed that preprin acts *via* IGF-II receptor, but receptors dedicated to preptin have not been indicated yet.

### Effect of Energy Homeostasis and Feeding

The available clinical data indicate that the plasma concentration of preptin was significantly higher in obese and overweight patients in comparison with healthy people and that it was positively correlated with BMI as well as waist circumference (Ozkan et al., 2013; El-Eshmawy and Abdel Aal, 2015). Nevertheless, it was also shown that, in morbidly obese patients, 6 months following bariatric surgery, the preptin level increased with borderline significance compared with the value before the operation (Glück et al., 2019). Moreover, postoperative analysis of patients showed that excess body weight and BMI were independently associated with preptin concentration, but were not significant to preptin when all subject characteristics were analyzed together (Glück et al., 2019).

### Effect on Glucose Homeostasis

**FIGURE 2:**
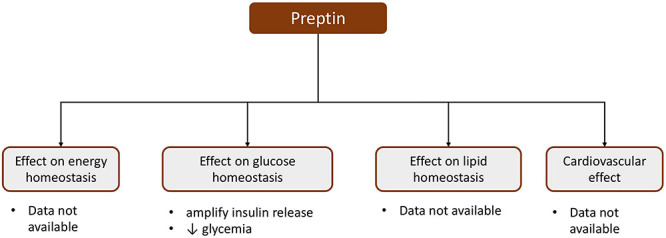
Schematic presentation of the effect of preptin.

Experimental studies have shown that preptin intensifies glucose-mediated insulin release, but does not initiate secretion (Figure 2) (Buchanan et al., 2001; Cheng et al., 2012). *In vitro* studies performed on MIN6 cells suggest that the effect of preptin on insulin release is mediated by the insulin-like growth factor II receptor (IGF2R), which activates phospolipidase C (PLC), protein kinase C (PKC), and Ca^2+^, and enhances insulin output under high glucose conditions (Cheng et al., 2012). A study conducted by Buchanan et al. (2001) on isolated, perfused rat pancreas showed that preptin can be a physiological amplifier of glucose-mediated insulin secretion, because the second phase of glucose-mediated insulin secretion increased, while anti-preptin immunoglobulin infusion caused a decrease in the first and second phases of insulin secretion. Additionally, it was presented that preptin i.v. infusion in fasted Wistar rats did not modify the glucose level, but it significantly decreased glycemia and increased the insulin level during glucose load (Cheng et al., 2012).

Research on humans has proven the significance of elevated preptin values in patients with metabolic disorders such as type 1 and 2 diabetes mellitus (DM) (Yang et al., 2009; Abd El Dayem et al., 2015), impaired glucose tolerance (IGT) (Bu et al., 2012), polycystic ovary syndrome (PCOS) (Celik et al., 2011; Mierzwicka et al., 2018), and gestational diabetes mellitus (GDM) (Aslan et al., 2011). Additionally, it was shown that higher preptin plasma concentrations in DM2 patients were positively correlated with higher diastolic blood pressure, triglyceride (TG), total cholesterol (TC), high-density lipoprotein (HDL-C), free fatty acids (FFA), 2-h blood glucose after glucose overload, hemoglobin A1c (HbA1c), and HOMA-IR (Yang et al., 2009).

A few studies on women with PCOS have found significantly higher blood concentrations of preptin (Celik et al., 2011, 2018; Bu et al., 2012; Mierzwicka et al., 2018; Şentürk et al., 2018). However, it is still unclear whether higher blood concentrations of preptin in women with PCOS were dependent on fasting insulin, glucose tolerance, and HOMA-IR (Yang et al., 2009; Celik et al., 2011; Bu et al., 2012).

To our knowledge, there is no literature or related data on the effect of preptin on lipid homeostasis.

### Cardiovascular Effect

Clinical trials have shown that lower blood concentrations of preptin, which were negatively correlated with carotid artery intima-media thickness, were observed in patients with primary hypertension and patients diagnosed with carotid atherosclerosis plaques (Cai et al., 2018). Additionally, systolic blood pressure (SBP) was independently linked with the plasma preptin level, which indicates its involvement in hypertension development (Cai et al., 2018). Furthermore, Abd El Dayem et al. (2015) reported a positive preptin correlation with SBP in the DM2 group. In DM2 patients, Yang (Yang et al., 2009) showed a positive preptin correlation with diastolic blood pressure (DBP), while Bu et al. (2012) also confirmed a positive preptin correlation with SBP and DBP in women with PCOS.

Wang et al. (2020) discovered that preptin single gene polymorphisms (rs1003483 and rs1004446 only) are relevant in patients suffering from coronary artery disease and hypertension. They reported that the rs1003483 genotype of patients with coronary artery disease is crucial in insulin secretion, whereas the rs1004446 genotype alters the TG level (Wang et al., 2020). There are other findings indicating that elevated preptin concentration is a coronary calcification predictor (a characteristic element of CAD) (Banerjee et al., 2020).

### Preptin and the Gut-Brain Axis

Preptin indirectly impacts the GBA by the influence on the enhancement of glucose-dependent insulin secretion (Buchanan et al., 2001, 2013; Cheng et al., 2012). It has been shown that the GBA is involved in the regulation of glycemia through detection of blood glucose levels by the enteric nervous system and the vagus nerve, which then sends an efficient efferent neural message to the peripheral organs, such as the pancreas, to induce insulin secretion and inhibit glucagon secretion (Schertzer and Lam, 2021). In addition, it has been proven that insulin, along with leptin, reaching the Arc nuclei of the hypothalamus mainly as humoral signals, creates a long-term energy response (Wilson and Enriori, 2015; Bauer et al., 2016; Sutton et al., 2016).

## Adropin

Secreted adropin (Adr) is a 43-amino acid peptide that is encoded by the Energy Homeostasis Associated (*Enho*) gene and is a final result of proteolytic cleavage of adropin 76 amino acid precursor (Kumar et al., 2008). Adropin is expressed in the brain [especially the dorsal premammillary nucleus of the hypothalamus (Kumar et al., 2008), PVN (Loewen and Ferguson, 2017), VMH (Butler et al., 2019), the thalamic nuclei (Kumar et al., 2008)], and in the medulla (including the dorsal vagal complex and the AP) (Kumar et al., 2008) (Table 3). It is also expressed peripherally in the rat in the liver, heart (Lovren et al., 2010), lung, kidneys, muscles, pancreas (Aydin et al., 2013b), and peripheral blood mononuclear cells (Kumar et al., 2008), and in non-human primates in the ileum and endocrine glands (Butler et al., 2019). The *Enho* transcript as well as the adropin protein were also confirmed in the human brain, liver (Kumar et al., 2008), umbilical vein, and coronary artery endothelial cells (Lovren et al., 2010). To date, the adropin receptor has not been definitively identified. Some researchers believe that the biological effects of adropin are obtained *via* direct binding to the G protein-coupled receptor GPR19 (Stein et al., 2016; Rao and Herr, 2017; Thapa et al., 2018) but a recently published article indicated that adropin does not interact with GPR19 (Foster et al., 2019). However, adropin can also bind to a brain-specific membrane-bound protein regulating motor coordination and physical activity *via* the NB-3/Notch signaling pathway in the brain (Wong et al., 2014).

**TABLE 3:** Expression of the adropin and GPR19 in humans, non-human primates, and rodents.

	**Expression in human**		**Expression in non-human primates**		**Expression in rat**		**Expression in mice**	
	**Peptide**	**Receptor**	**Peptide**	**Receptor**	**Peptide**	**Receptor**	**Peptide**	**Receptor**
Nervous system	Brain	N/A	Amygdala, Arc, cerebellum, dorsomedial hypothalamus, habenula, lateral globus pallidus, medial globus pallidus, LHA, mammillary bodies, olfactory bulb, pons, preoptic area, prefrontal cortex, PVN, suprachiasmatic nuclei, supraoptic nucleus, substantia nigra, thalamus, visual cortex, VMH	N/A	Brain (vascular area, pia mater, neuroglial cell, PVN, supraoptic nucleus, circumventricular organs, cerebral cortex, and neurons), cerebellum (neuroglial cells, Purkinje cells, vascular areas, and granular layer), brain microvascular endothelial cells (RBE4)	Basal and rostral hypothalamus, PVN, supraoptic nucleus	Medial habenula, hypothalamus, dorsal premamillary nucleus of the hypothalamus, medial septal complex (medial septum and the nucleus of the diagonal band), thalamus (the posterior thalamic nuclear group, the lateral and medial geniculate nuclei, and the paraventricular, anterodorsal, ventral posterolateral, and ventral posteromedial thalamic nuclei), substantia nigra pars compacta, interpeduncular nucleus, red nucleus, periaqueductal gray, and median raphe, pontine nuclei, reticulotegmental nucleus of the pons, medial vestibular nucleus, prepositus nucleus, facial nucleus, inferior olive, lateral reticular nucleus, dorsal vagal complex, area postrema, and cuneate nucleus.	Brain
Peripheral tissues	Liver, salivary glands, stomach (human gastric carcinoma), human umbilical vein endothelial cells, human coronary artery endothelial cells, human aortic endothelial cells, skeletal muscles feed arteries, THP1 monocytes, THP1 monocyte-derived macrophages, human aortic smooth muscle cells, small intestine Paneth cells	Cardiac cells, THP1 monocytes, THP1 monocyte-derived macrophages, human umbilical vein endothelial cells, human aortic endothelial cells, human aortic smooth muscle cells	Liver, kidneys, esophagus, stomach, duodenum, ileum, ascending colon, descending colon, cecum, smooth muscles, lung, prostate, testes, skin, white adipose tissue, bone marrow, spleen, heart, aorta, skeletal muscles, pituitary glands, pineal gland, adrenal glands, pancreas, thyroid gland	N/A	Kidneys (glomerulus, peritubular interstitial cells, and peritubular capillary endothelial cells, the thin limb of the loop of Henle in the medulla), heart (endocardium, myocardium, and epicardium), liver (sinusoidal cells, hepatocytes, Kupffer cells), and pancreas (serous acini), skeletal muscles, white adipose tissue, brown adipose tissue	Heart, white adipose tissue, brown adipose tissue, pancreas	Liver, skeletal muscles, white adipose tissue, brown adipose tissue, spleen, intestine, lung	N/A

*Arc, arcuate nucleus; LHA, lateral hypothalamic area; N/A, not available; PVN, paraventricular nucleus; VMH, ventromedial hypothalamus.*

### Effect on Energy Homeostasis and Feeding

Adropin expression appears to be influenced by the type of diet and the duration of its application. It has been shown that feeding on a high-fat diet for 4 weeks caused an increase, whereas feeding on a high-fat diet for 3 months caused a decrease in hepatic *Enho* expression in C57BL/6J (B6) mice (Kumar et al., 2008; Banerjee et al., 2020). Similarly, a downregulation in liver *Enho* mRNA expression was observed in mice with genetically induced obesity due to a deficiency of the leptin receptor (*Lep^*ob*^/Lep^*ob*^*) or the melanocortin receptor (lethal yellow *A^*y*^/a, Mc3r*^–/–^, and *Mc4r*^–/–^) (Kumar et al., 2008). Additionally, transgenic B6 mice overexpressing adropin (AdrTG), fed a high-fat diet at 8–14 weeks of age, revealed significantly reduced weight gain due to the reduction of fat mass compared with the control (Kumar et al., 2008). Later studies confirmed that adropin knockout mice (AdrKO) on the C57BL/6J background, maintained for 8 weeks on a high-fat diet, showed weight gain compared with the control mice (wild-type mice: WT) (Ganesh Kumar et al., 2012). In turn, an increase in body weight was observed due to excessive proliferation of adipose tissue in AdrKo mice on a standard diet (50% increase compared with wild-type: WT) without altering food consumption or energy expenditure (Ganesh Kumar et al., 2012). Whereas, Chen et al. (2017) demonstrated that AdrKO and heterozygous carriers of the null allele (C57BL/6- Enhotm1.1Butl, referred to below as AdrHET), which were on a high-fat diet for 8 weeks, had a significantly higher body weight than WT mice. Based on the studies by Ghoshal et al. (2018), it can be assumed that the peak of *Enho* expression in the liver during the daily cycle occurs during the period of maximum nutrition, while the lowest expression during the resting phase (Ghoshal et al., 2018).

Moreover, Ganesh Kumar et al. (2012) revealed that higher plasma adropin concentrations were observed in C57BL/6J (B6) mice on a standard diet, whereas feeding the mice on a high-fat diet for 31 days or fasting them resulted in a reduction in plasma adropin levels. Similarly, lower plasma levels of adropin were observed in rhesus macaques maintained for 3 months on a high-fructose diet, which may show adropin as a potential marker of weight gain and metabolic disorders (Butler et al., 2019). Moreover, it was shown that fasting also influences adropin circulation. In female C57BL/6 mice, the adropin level was significantly decreased after 4 weeks of a caloric restriction (CR) diet, while 74 weeks of CR resulted in a concentration five times higher (Kuhla et al., 2014).

Clinical studies have shown that adropin concentration in plasma can be closely related to the nutrient composition in the diet. The plasma adropin level is positively correlated with fat consumption in women, but not in men (St-Onge et al., 2014). Furthermore, clinical studies have revealed lower plasma adropin concentrations in overweight and obese patients, which can indicate significant negative adropin association with BMI (Butler et al., 2012; Sayin et al., 2014; Wu et al., 2014; Yu et al., 2014; Altincik and Sayin, 2015; Gu et al., 2015; Yosaee et al., 2017; Zhang et al., 2017; Zang et al., 2018; Chen et al., 2019; Yuan et al., 2020). Additionally, the concentration of adropin in plasma was also lower in females (age-adjusted), in elderly people, and patients with more than two risk factors of metabolic syndrome (MetS) and with MetS itself (Butler et al., 2012; Yosaee et al., 2017). Interestingly, it was shown that the plasma concentration of adropin did not change due to weight loss in obese patients, but the weight loss was dependent on baseline values (Stevens et al., 2016). Moreover, the Roux-en-Y gastric bypass (RYGB) procedure resulted in raised plasma concentrations of adropin, hitting the peak point 3 months after the operation and regressing to the baseline at 12 months after the operation (Butler et al., 2012). Similarly, Glück et al. (2019) reported that the adropin plasma concentration level increased at 6 months after bariatric surgery compared with the baseline, where the increase was intensified in younger obese patients with lower baseline adropin levels.

### Effect on Glucose Homeostasis

**FIGURE 3:**
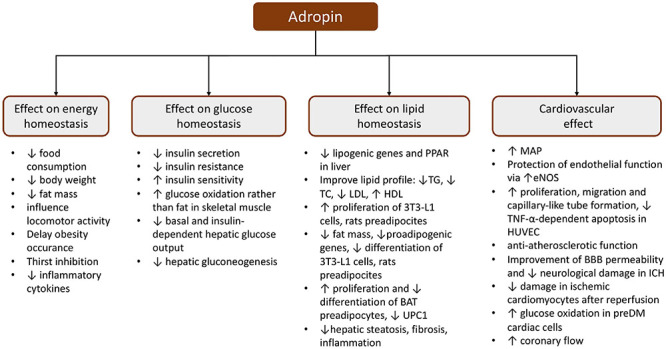
Schematic presentation of the pleiotropic effect of adropin. BAT, brown adipose tissue; BBB, blood–brain barrier; eNOS, endothelial nitric oxide synthase; HDL, high-density lipoprotein; HUVEC, human umbilical vein endothelial cells; ICH, intracerebral hemorrhage; LDL, low-density lipoprotein; MAP, mean arterial pressure; PPAR, peroxisome proliferator-activated receptor; TC, total cholesterol; TG, triglyceride; TNF-α, tumor necrosis factor α; UPC1, uncoupling protein 1.

The *Enho* final product influences glucose homeostasis (Figure 3). It has been shown that adropin can directly affect the cells of the pancreas. Billert et al. (2020) recently reported that adropin decreased insulin mRNA expression and leads to the reduction of glucose-dependent insulin secretion *via* the inhibition of cyclic adenosine monophosphate (cAMP) synthesis in rat insulin-producing INS-1E cells as well as in pancreatic islets isolated from male Wistar rats. However, adropin did not influence INS-1E cell proliferation (Billert et al., 2020).

Numerous studies have proven that a high level of plasma adropin reduces insulin resistance and glucose intolerance and that adropin can stimulate glucose consumption and improve carbohydrate and energy metabolism (Kumar et al., 2008; Ganesh Kumar et al., 2012; Gao et al., 2014; Akcilar et al., 2016b). Kumar et al. (2008) showed that 14 days i.p. injection of adropin in C57BL/6J mice fed on a high-fat diet caused not only a reduction in food intake linked with weight loss, but also decreased hyperinsulinemia without affecting the blood glucose level, and it also regressed hepatic steatosis by the upregulation of genes providing TG synthesis and absorption (Kumar et al., 2008). Moreover, this same therapy, lasting 2 days, significantly decreased the fasting insulin and HOMA-IR, which indicates adropin amelioration of glucose and hepatic lipid homeostasis, which was independent of weight loss or food consumption (Kumar et al., 2008). Interestingly, transgenic AdrKO B6 mice showed hypertriglyceridemia and an impaired suppression in endogenous glucose production in hyperinsulinemic-euglycemic clamp conditions, which indicates insulin resistance (Ganesh Kumar et al., 2012). Whereas, Chen et al. demonstrated that a high-fat diet challenged AdrKO B6 mice and revealed elevated hepatic glucose production in the case of whole-body insulin resistance (Chen et al., 2020a). Further studies revealed that AdrKO B6 mice fed on a standard chow diet not only had their glycemia altered but also expanded adipocyte infiltration of the exocrine pancreas tissue, which is distinctive for fatty pancreas (FP) disease (Chen et al., 2017). AdrKO and AdrHET B6 mice finishing 8 weeks on a high-fat diet exhibited increased insulin levels, but only AdrKO had hypertriglyceridemia (Chen et al., 2017). It has also been shown that transgenic AdrKO B6 mice revealed a preferential stimulation of fat oxidation over glucose in muscles (Gao et al., 2014). Moreover, higher plasma adropin concentrations and higher liver *Enho* mRNA expression were observed in Sprague-Dawley rats with type 1 diabetes (Kuo et al., 2020). Additionally, a study in Wistar rats with hyperlipidemia induced by a high-fat diet showed that 10 days of i.p. infusion of adropin contributed to reduced glycemia, insulinemia, and HOMA-IR which may indicate an improvement in carbohydrate hemostasis (Akcilar et al., 2016a). Similarly, 10 days of i.p. adropin infusion in high-fat diet DM2 Wistar rats resulted in an improvement of carbohydrate metabolism (lower glycemia, HbA1c, and HOMA-IR, and higher HOMA-B and serum insulin levels) and a reduction of inflammatory cytokines (Akcilar et al., 2016b). In turn, increased adropin synthesis in the pancreatic, liver, kidney, heart, and brain tissues was observed in Wistar rats developing type 1 diabetes (Aydin et al., 2013b). Furthermore, insulin and phloridzin treatment in diabetic Sprague-Dawley rats decreased serum adropin concentration, causing downregulation of *Enho* and STAT3 expression in the liver in comparison with the control rats (Kuo et al., 2020).

Recently, the possibility of using adropin as a therapeutic agent to improve glucose tolerance has often been emphasized. Thapa et al. showed that the administration of synthetic adropin for 3 days to C57BL/6J mice on a high-fat diet contributed to the improvement of glucose tolerance by lowering fasting glucose levels and they also showed that the reduction of basal and insulin-dependent glucose production in the liver was caused by downregulation of lipid transporters (CD36; carnitine palmitoyltransferase Ia, CPT1a) and gluconeogenic enzymes (G6PC) (Thapa et al., 2019a). Additionally, Gao et al. indicated that treatment with adropin improves insulin sensitivity in the liver of DIO B6 mice by an increase in IRS1/2-Akt phosphorylation leading to lower GSK3 activity, promotion of glycogen synthesis, and lower FoxO1 transcript, which upregulates GCK and downregulates G6PC and PCK1 (Gao et al., 2019). In addition, adropin contributed to the attenuation of glucose production in hepatocytes *via* cAMP/PKA signaling (Gao et al., 2019).

Clinical studies have shown lower adropin serum concentration in GDM women, obese adolescents with insulin resistance, DM2, and DM1 children (Celik E. et al., 2013; Sayin et al., 2014; Wu et al., 2014; Altincik and Sayin, 2015; Zhao et al., 2015; Hu and Chen, 2016; Chen et al., 2017; Chen et al., 2019; Polkowska et al., 2019). In DM2 patients diagnosed with fatty pancreas, downregulation of adropin was observed (Chen et al., 2017). Moreover, Zang et al. (2018) indicated that obese DM2 subjects presented a greater reduction in adropin levels when compared with obese healthy controls. Additionally, in DM1 children, the adropin concentration depended on the duration of the disease, with the lowest level recorded in newly diagnosed patients in comparison with patients treated for over 10 years (Polkowska et al., 2019).

### Effect on Lipid Homeostasis

The available data indicate that adropin plays an important role in the metabolism of white adipose tissue (Jasaszwili et al., 2019, 2020). Evidence for the above observation was provided by *in vitro* studies on mouse fibroblast cells (3T3-L1) and rat primary preadipocytes, which both, when treated with adropin, increased their proliferation through ERK1/2 and Akt-dependent signal transduction and reduced their lipid accumulation and expression of proadipogenic genes (*Pparγ, Fabp4*, *C/ebpα*), ultimately reducing their differentiation into mature adipocytes (Jasaszwili et al., 2019). Similarly, adropin stimulated the proliferation of brown adipose tissue (BAT) preadipocytes in Wistar rats *via* the Akt-dependent pathway, but impaired preadipocyte differentiation *via* downregulation of the adipogenic genes (*C/ebpα*, C*/ebpβ*, *Pgc1α*, *Pparγ*, and *Prdm16*), and lowered the expression of UPC1 mRNA and UPC1 protein (Jasaszwili et al., 2020). In addition, the same researchers showed that adropin not only reduced the fat volume in BAT but also enhanced the outflow of glycerol and FFA promoting hormone sensitive lipase activity, simultaneously (Jasaszwili et al., 2020).

It was shown that adropin also reduced the expression of hepatic lipogenic genes and adipose tissue peroxisome proliferator-activated receptor gamma in B6 mice (Kumar et al., 2008; Gao et al., 2019). Additionally, hyperlipidemic Wistar rats treated 10 days with adropin (i.p.) showed an insignificant decrease in TG and total cholesterol, a significant reduction in body weight, and low-density lipoprotein (LDL), and an increase in HDL (Akcilar et al., 2016a). Similarly, DM2 Wistar rats on a high-fat diet treated with adropin for 10 days revealed an improvement in lipid metabolism, as evidenced by lower serum concentrations of TC, TG, LDL-C as well as higher concentrations of HDL (Akcilar et al., 2016b). Whereas, studies carried out on transgenic B6 mice overexpressing adropin (Adr-Tg) on a high cholesterol diet did not confirm an adropin protective function against hypercholesterolemia and arteriosclerosis, suggesting that adropin does not influence cholesterol uptake, nor circulation-synthesis clearance (Ghoshal et al., 2018). Furthermore, transgenic AdrKO mice, fed either on a western diet (WD) or on a methionine-choline deficient diet (MCD), developed more advanced hepatic microsteatosis, inflammation, and fibrosis irrespective of the diet and presented higher reactive oxygen species (ROS) levels (Chen et al., 2019). Similarly, in C57BL/6J wild-type mice, diet-induced non-alcoholic steatohepatitis (NASH), a reduction in adropin serum levels was reported and the downregulation of liver and adipose *Enho* mRNA (Chen et al., 2019). In turn, the administration of high doses of exogenous bioactive adropin to these mice caused ameliorated NASH progression, possibly through activation of the nuclear factor erythroid 2-related factor 2 (Nrf2) pathway (Chen et al., 2019). Further studies showed that adropin therapy in combination with exercise was effective in alleviating NASH progression in C57BL/6J mice fed on a high-fat diet or an MCD diet, possibly due to an adropin anti-inflammatory effect *via* a reduction in ROS levels, which suppresses NLR family pyrin domain containing 3 (NLRP3) activation (Yang et al., 2021).

Clinical studies have shown significantly lower blood levels of adropin in overweight and obese patients which were negatively correlated with TG levels and positively correlated with HDL (Zang et al., 2018; Yuan et al., 2020). Moreover, the concentration of adropin in plasma was inversely correlated with LDL in men, while a similar relationship was not found in women (Ghoshal et al., 2018). Nevertheless, among Taiwanese adolescents, no changes in lipid profile with regards to adropin concentration were found (Chang et al., 2018). It was also found that adropin may play an important role in the pathophysiology of non-alcoholic fatty liver disease (NAFLD) (Sayin et al., 2014; Kutlu et al., 2019; Chen et al., 2020b). Obese adolescents with NAFLD had decreased adropin in comparison with the obese adolescent without NAFLD and with the control, and it was concluded that decreased adropin was an independent risk factor of NAFLD in this group (Sayin et al., 2014). Studies conducted on adults with NAFLD showed significantly decreased serum adropin levels, which was inversely related to serum insulin, HOMA-IR, TC, and TG levels (Kutlu et al., 2019). Further research reported a negative correlation between the adropin level and the severity of histopathological changes in NAFLD, such as steatosis, inflammation, and fibrosis (Chen et al., 2020b).

### Cardiovascular Effect

Numerous studies indicate that adropin influences cardiovascular system function and participates in the pathogenesis of several cardiovascular diseases (Lovren et al., 2010; Topuz et al., 2013; Wu et al., 2014; Gu et al., 2015; Zhao et al., 2015, 2016; Gulen et al., 2016; Ertem et al., 2017; Oruc et al., 2017; Bolayır et al., 2018; Sato et al., 2018; Altamimi et al., 2019; Thapa et al., 2019b). Firstly, adropin determines the condition of the endothelial cells (EC). It was reported that adropin stimulated cell proliferation, migration, capillary-like tube formation, and decreased apoptosis depending on tumor necrosis factor α (TNF-α) in human umbilical vein endothelial cells (HUVECs) (Lovren et al., 2010). Furthermore, the same researchers demonstrated that adropin caused an improvement in endothelial barrier permeability by the upregulation of NO synthase expression through the VEGFR2-phosphatidylinositol 3-kinase-Akt and VEGFR2-extracellular signal regulated kinase 1/2 pathways (Lovren et al., 2010). Further *in vitro* studies also performed in HUVECs, as well as in human aortic smooth muscle cells (HASMCs) showed that adropin treatment inhibited TNF-α-induced THP1 monocyte adhesion to HUVECs, and in turn, adropin suppressed migration and proliferation in HASMCs without inducing apoptosis *via* ERK1/2 and Bax downregulation, and PI3K/Akt/Bcl2 upregulation (Sato et al., 2018). Based on the above observations, it can be assumed that adropin may have anti-inflammatory and antiatherosclerotic effects in blood vessels, which was also confirmed by *in vivo* studies. It has been shown that chronic administration of adropin to ApoE-knockout (Apoe^–/–^) mice attenuated the development of atherosclerotic lesions in the aorta, with reduced intra-plaque monocyte/macrophage infiltration and smooth muscle cell content (Sato et al., 2018). Moreover, it was proven that adropin therapy contributed to improved angiogenic potential by increasing the blood flow and density of capillary in male BALB/c and homozygous NOS3, EC knockout Nos3tm1Unc/J mice in whom unilateral hindlimb ischemia had been performed (Lovren et al., 2010).

In addition, based on the available data, it may be concluded that adropin has a beneficial effect on the function and metabolism of the heart (Altamimi et al., 2019; Thapa et al., 2019b; Wu et al., 2019). It was shown that treatment of cardiomyocyte H9c2 cells, subjected to ischemic and reperfusion conditioning with adropin, had antioxidant and anti-inflammatory effects, consequently reducing myocardial apoptosis (Wu et al., 2019). Moreover, treatment of C57Bl/6 mice hearts with adropin was found to result in the inhibition of fatty acid oxidation, accompanied by a robust stimulation of glucose oxidation (Altamimi et al., 2019). Consequently, higher cardiac work was noticed, which was accompanied by improved cardiac efficiency and enhanced insulin signaling in adropin-treated mouse hearts (Altamimi et al., 2019). It appears that adropin may influence myocardial metabolism through increased activation of downstream cardiac insulin signaling, which was manifested as a reduction in the inhibitory phosphorylation of pyruvate dehydrogenase (PDH), the major enzyme of glucose oxidation, PDH kinase 4, and the insulin-signaling inhibitory phosphorylation of JNK (p-T183/Y185) and IRS-1 (p-S307) (Altamimi et al., 2019).

Moreover, it was proven that adropin influences the permeability of the blood-brain barrier (BBB) (Yang et al., 2016). In ischemic conditions, rat brain endothelial cells 4 (RBE4), pretreated with adropin, presented a dose-dependent reduction of permeability through the blockage of the ROCK-MLC2 signaling pathway (Yang et al., 2016). Furthermore, a study conducted in CD1 mice with intracerebral hemorrhage (ICH) revealed that intranasally inhaled recombinant human adropin diminished BBB injury, as well as reducing neurological damage (edema) and dysfunctions *via* the Notch1/Hes1 signaling pathway (Yu et al., 2017).

Numerous clinical studies revealed that decreased plasma adropin concentration is related to CVDs, such as hypertension (Gu et al., 2015; Gulen et al., 2016; Bolayır et al., 2018), cardiac syndrome X (Celik A. et al., 2013), CAD (Wu et al., 2014; Zhao et al., 2015, 2016; Ertem et al., 2017), and conditions linked to endothelial dysfunctions (Topuz et al., 2013; Oruc et al., 2017).

In particular, lower serum adropin levels were reported in patients with acute myocardial infarction (AMI) compared with stable angina pectoris (SAP) patients or controls, which may indicate adropin as an independent predictor for the presence of AMI in CAD patients (Yu et al., 2014). Similarly, patients with non-ST segment elevation myocardial infarction (NSTEMI) and a high SYNTAX score (used for assessing the severity of CAD) had significantly lower serum adropin levels compared with NSTEMI patients with a low SYNTAX score (Ertem et al., 2017). It was also found that low serum adropin concentrations were an independent predictor for a high SYNTAX score (Ertem et al., 2017). Additionally, in patients with enzyme-positive acute coronary syndrome (EPACS), significant increases in serum and salivary adropin levels were observed up to 6 h after the event and then began to decline (Aydin et al., 2017). Based on the above results, it can be assumed that the measurement of adropin concentration in serum and saliva samples may be a new potential marker for diagnosing EPACS (Aydin et al., 2017). Furthermore, there is evidence that low adropin serum may be a prognostic marker for stable CAD (SCAD) and the severity of coronary arteriosclerosis (Zhao et al., 2015). Importantly, it was proven that low concentrations of adropin in the serum may be an independent indicator of coronary arteriosclerosis and its severity in DM2 and non-DM2 patients (Wu et al., 2014; Zhao et al., 2016). Significantly decreased serum adropin concentrations were also found in permanent atrial fibrillation (AF) patients compared with persistent and paroxysmal AF patients and with healthy controls (Wang et al., 2019). In addition, it was suggested that serum adropin in AF patients were negatively correlated with BMI, SBP, and left atrial diameter (Wang et al., 2019).

Numerous studies have revealed significantly lower blood levels of adropin in lesions of the cardiovascular system, accompanied by endothelial dysfunction of the blood vessels (Motz et al., 1991; Celik A. et al., 2013; Topuz et al., 2013; Oruc et al., 2017; Kwon et al., 2019). One of the diseases associated with endothelial dysfunction is cardiac syndrome X (CSX) A significant decrease in serum adropin concentration in patients suffering from CSX has been demonstrated (Celik A. et al., 2013). Lower blood levels of adropin were also characteristic of endothelial damage in the course of DM2 and MetS (Topuz et al., 2013; Oruc et al., 2017).

Clinical studies have shown that in the course of heart failure, a significant increase in plasma adropin concentration is observed, which was positively correlated with the New York Heart Association (NYHA) functional classification scale, brain natriuretic peptide, and BMI as well as negatively correlated with the left ventricular ejection fraction (LVEF) (Lian et al., 2011).

The results of studies on the role of adropin in hypertension appear to be inconclusive. Studies on obese children have shown that serum levels of adropin did not correlate with changes in blood pressure, although they were characteristic of obesity (Altincik and Sayin, 2015). On the other hand, in adult hypertensive patients, lower plasma levels of adropin were observed, which was negatively correlated with SBP, DBP, and endothelin 1 (Gu et al., 2015). Gulen et al. (2016) also reported a decreased adropin level in untreated hypertension patients, which was not associated with acute hypertensive target organ injury. Further analysis of ambulatory 24-h monitoring exposed a negative correlation between nighttime BP and adropin concentration with a lower level in non-dipper subjects in comparison with dipper and normotensive patients (Bolayır et al., 2018). In contrast, in patients with untreated obesity-induced essential hypertension, higher plasma levels of adropin were found, which further increased after 12 weeks of antihypertensive treatment with amlodipine or valsartan (Çelik et al., 2015).

### Adropin and the Gut-Brain Axis

It appears that adropin may play a role in the gut-brain axis as it has been highly expressed in structures involved in food intake regulation both in the CNS (hypothalamic nuclei) and peripherally in the intestine and liver (Kumar et al., 2008; Loewen and Ferguson, 2017; Butler et al., 2019). Experimental and clinical studies have shown that the expression of adropin in the brain and the plasma is related to the composition of nutrients in the diet (Kumar et al., 2008; Ganesh Kumar et al., 2012). Rhesus macaques with diminished adropin levels were characterized by higher fasting glucose and leptin levels (Butler et al., 2019) and an analysis was performed that showed that leptin, HDL-C, ApoA1, and ApoC3 are highly significant predictors of plasma adropin concentrations irrespective of diet, after fructose challenging only TG ApoB (Butler et al., 2019). Moreover, it has been proven that adropin is directly involved in the regulation of insulin metabolism, one of the most important humoral factors regulating the activity of hunger and satiety centers in the brain (Wilson and Enriori, 2015; Sutton et al., 2016; Billert et al., 2020).

## Conclusion

The presented data obtained in recent years reveal that 26/43RFa neuropeptides, preptin, and adropin play an important role in the pathogenesis of obesity, metabolic syndrome, and cardiovascular diseases. Moreover, it appears that the above peptides influence GBA and simultaneously take part in crosstalk between metabolic and cardiovascular diseases and the central nervous system. The peptides described in this study appear to have potential pharmacological utility in the treatment of obesity comorbidities that can lead to better control of CVD and metabolic disease, and also directly influence a person’s obese state. Despite growing data from the research, further experimental and clinical studies are necessary.

## Author Contributions

MC and AC-J created the concept of the article. MC performed the literature search, data analysis, and drafted the manuscript. MC created the figures. AC-J, KC, and MC critically revised the work and prepared the final version of the manuscript. All authors accepted the final version of the manuscript.

## Conflict of Interest

The authors declare that the research was conducted in the absence of any commercial or financial relationships that could be construed as a potential conflict of interest.

## Publisher’s Note

All claims expressed in this article are solely those of the authors and do not necessarily represent those of their affiliated organizations, or those of the publisher, the editors and the reviewers. Any product that may be evaluated in this article, or claim that may be made by its manufacturer, is not guaranteed or endorsed by the publisher.

## Abbreviations

α-MSH: α-melanocyte -stimulating hormone
Adr: adropin
AF: atrial fibrillation
ACSL1: long-chain-fatty-acid-CoA ligase 1
AgRP: agouti-related peptide
Akt: serine/threonine-protein kinase
AMI: acute myocardial infarction
ANS: autonomic nervous system
AP: area postrema
ApoE: apolipoprotein E
Arc: arcuate nucleus
B6 mice: C57BL/6J mice
BBB: blood-brain barier
BMI: body mass index
BAT: brown adipose tissue
CAD: coronary artery disease
cAMP: cyclic adenosine monophosphate
CART: cocaine and amphetamine regulated transcript
CCK: cholecystokinin
CD36: cluster of differentiation 36
C/ebp α: CCAAT/enhancer-binding protein alpha
C/ebp β: CCAAT/enhancer-binding protein beta
CNS: central nervous system
CO: cardiac output
CPT1a: carnitine palmitoyltransferase Ia
CRH: corticotropin-releasing hormone
CR: caloric restriction
CVD: cardiovascular diseases
DBP: diastolic blood pressure
EC: endothelial cells
EECs: enteroendocrine cells
Enho: energy homeostasis associated
DIO: diet induced obesity
DM: diabetes mellitus
DMV: the dorsal nucleus of vagus nerve
DVC: dorsal vagal complex
EPACS: enzyme-positive acute coronary syndrome
ERK1/2: extracellular signal regulated kinases 1/2
Fabp4: fatty acid-binding protein 4
FATP1: long-chain fatty acid transport protein 1
FFA: free fatty acids
FP: fatty pancreas
FoxO1: forkhead box protein O1
GBA: gut-brain axis
GCK: glucokinase
GDM: gestational diabetes mellitus
GI: gastrointestinal
GLP-1: glucagon-like peptide-1
GLP-1R: glucagon-like peptide-1 receptor
GLP-2R: glucagon-like peptide-2 receptor
GPR19: G-protein coupled receptor 19
GPR103: G-protein coupled receptor 103
G6PC: glucose-6-phosphatase
GSK3: Glycogen synthase kinase 3
HbA1c: hemoglobin A1c
HDL-C: high density lipoprotein cholesterol
HCRTR1: hypocretin receptor 1
HF: heart failure
HASMCs: human aortic smooth muscle cells
HOMA-B: homeostasis model assessment of β-cell dysfunction
HOMA-IR: homeostatic model assessment for insulin resistance
HPA: hypothalamic-pituitary-adrenal axis
HR: heart rate
HUVECs: human umbilical vein endothelial cells
ICH: intracerebral hemorrhage
ICV: intracerebroventricular
(pro)IGF-II: (pro)insulin-like growth factor II
IGF2R: insulin-like growth factor II receptor
IGT: impaired glucose tolerance
i.p: intraperitoneal
ISO: isoproterenol
i.v.: intravenous
JNK: c-Jun NH2-terminal kinase
KO: knock out
LHA: lateral hypothalamic area
LPL: lipoprotein lipase
LV: left ventricle of heart
LDL-C: low density lipoprotein cholesterol
MAP: mean arterial pressure
MC4R: melanocortin 4 receptor
MCD: methionine-choline diet
MetS: metabolic syndrome
MIN6 cells: mouse insulinoma cells
MLC2: myosin light chain 2
NAFLD: nonalcoholic fatty liver disease
NASH: nonalcoholic steatohepatitis
NLRP3: NLR family pyrin domain containing 3
NPY: neuropeptide Y
Nrf2: nuclear factor erythroid 2-related factor 2
NSTEMI: non-ST segment elevation myocardial infarction
NTS: nucleus of tractus solitarius
OGTT: oral glucose tolerance tests
OXM: oxyntomodulin
PDH: pyruvate dehydrogenase
PCK1: phosphoenolpyruvate carboxykinase 1
PCOS: polycystic ovary syndrome
Pgc1 α: peroxisome proliferator activated receptor gamma coactivator 1-alpha
PI3K: phosphoinositide 3-kinases
PKA: protein kinase A
PKC: protein kinase C
PLC: phospolipidase C
POMC: proopiomelanocortin
PPAR- γ: peroxisome proliferator-activated receptor gamma
Prdm16: PR domain containing 16
PVN: paraventricular nucleus
PYY: peptide YY
RBE4: rat brain endothelial cells 4
ROCK: Rho-associated kinase
ROS: reactive oxygen species
RYGB: Roux-en-Y gastric bypass
SAP: stable angina pectoris
SBP: systolic blood pressure
STAT3: signal transducer and activator of transcription 3
SV: stroke volume
SVR: vascular resistance
TC: total cholesterol
TG: triglyceride
TNF- α: tumor necrosis factor α
TRH: thyrotropin-releasing hormone
UPC1: uncoupling protein 1
VEGFR2: vascular endothelial growth factor 2
VMH: ventromedial hypothalamus
WAT: white adipose tissue
WD: western diet
WHO: World Health Organization
WT: wild type.
